# Leucocytosis and Asymptomatic Urinary Tract Infections in Sickle Cell Patients at a Tertiary Hospital in Zambia

**DOI:** 10.1155/2020/3792728

**Published:** 2020-06-02

**Authors:** Taonga Musonda, Mildred Zulu, Mulemba Samutela, Annie Kalonda, Hamakwa Mantina, Pauline Okuku, Musalula Sinkala, Panji Nkhoma

**Affiliations:** ^1^Department of Biomedical Sciences, School of Health Sciences, University of Zambia, Lusaka, Zambia; ^2^Department of Pathology and Microbiology, School of Medicine, University of Zambia, Lusaka, Zambia; ^3^Department of Pathology and Microbiology, University Teaching Hospitals, Lusaka, Zambia

## Abstract

Sickle cell anaemia (SCA) is an inherited disease resulting from mutations in the *β*-globin chain of adult haemoglobin that results in the formation of homozygous sickle haemoglobin. It is associated with several complications including an altered blood picture and damage in multiple organs, including the kidneys. Kidney disease is seen in most patients with SCA and may affect glomerular and/or tubular function, thereby putting these patients at risk of urinary tract infections. However, there is a paucity of data on the prevalence of urinary tract infections (UTIs) among SCA patients in Zambia. This study aimed to determine the prevalence of UTIs and haematological and kidney function profiles among SCA patients at the University Teaching Hospitals, Lusaka, Zambia. This was a cross-sectional study conducted between April and July 2019 involving 78 SCA patients who presented at the UTH. Blood and midstream urine samples were collected from each participant using the standard specimen collection procedures. Full blood counts and kidney function tests were determined using Sysmex XT-4000i haematology analyser and the Pentra C200 by Horiba, respectively. Bacterial profiles of the urine samples were determined using conventional microbiological methods. We found that all the measured patients' haemoglobin (Hb) levels fell below the WHO-recommended reference range with a minimum of 5 g/dl, a maximum of 10.5 g/dl, and a mean of 8 ± 1 g/dl. Fifty percent of the participants had moderate anaemia, while the other 50% had severe anaemia. The minimum WBC count of the participants was 0.02 × 10^9^/L with a maximum of 23.36 × 10^9^/L and a mean of 13.48 ± 3.87 × 10^9^/L. Using the one-way analysis of variance test, we found no significant difference in mean WBC count and Hb concentration across various age-group categories that we defined. Bacteriuria was found in 25% of participants. The most common bacterial isolates were *Staphylococcus aureus* (32%) and coagulase-negative *Staphylococci* (32%). *Klebsiella pneumoniae* was 16%. We found no significant association between bacterial isolates and white blood cell count, age groups, sex, and anaemia severity *p* = 0.41. None of the participants were diagnosed with kidney disease. There was a high prevalence of asymptomatic UTIs among SCA patients at UTH, which, when coupled with the marked leukocytosis and anaemia, may negatively impact the clinical outcome of the patients. Therefore, we recommend close monitoring of sickle cell patients in Zambia for such conditions to improve patients' outcomes.

## 1. Introduction

Sickle cell anaemia (SCA) is an inherited disease resulting from mutations in the *β*-globin chain of adult haemoglobin that results in the formation of homozygous sickle haemoglobin (HbSS). In SCA, red blood cells deform into a sickle shape and damage cell membranes [1]. The disease is associated with significant and costly long-term complications and reduced life expectancy [1–3]. It is also generally associated with higher mortality in both children and adults [4, 5]. Estimates suggest that every year approximately 300,000 infants are born with sickle cell anaemia and that this number could rise to 400,000 by 2050 [6]. Although the burden of SCA is truly global, more than 75% of the global burden of SCA occurs in sub-Saharan Africa [6]. The fundamental pathological processes in SCA include blood vessel occlusion, erythrocyte sickling, and recurrent infections due to immune compromise [7–9].

The disease is associated with various complications ranging from an altered blood picture to complications such as leukocytosis, increased platelets, and decreased hematocrit and haemoglobin [10] and multiple organ disorders, including those of the kidney. Kidney disease is seen in most patients with SCA and may affect glomerular and/or tubular function [11]. Abnormalities in the function of the kidney are believed to increase the asymptomatic bacteriuria rate, which leads to UTIs in SCA patients. It is for this reason that sickle cell anaemia has some effect on the type and distribution of UTI-causing bacteria. These UTIs are either symptomatic with bacteriuria and associated with symptoms such as dysuria, pyuria, and frequent urination or asymptomatic (no acute symptoms) with bacteriuria [12]. The classical definition of asymptomatic urinary tract infection also called asymptomatic bacteriuria is the isolation of a specified quantitative count of bacteria in an appropriately collected urine specimen obtained from a person without symptoms or signs referable to urinary infection. While that for acute uncomplicated urinary tract infection is symptomatic bladder infection characterised by frequency, urgency, dysuria, or suprapubic pain in a woman with a normal genitourinary tract, and it is associated with both genetic and behavioural determinants [13]. Delay in detecting and thereby institution of appropriate treatment may lead to the formation of scars and subsequent renal impairment in sickle cell anaemia (SCA) individuals which may lead to precipitation of crisis and fatal septicaemia [14].

However, there is a paucity of data on the prevalence of both symptomatic and asymptomatic urinary tract infections among SCA patients in Zambia. Therefore, this study aimed to determine the prevalence of UTIs and haematological and kidney function profiles among SCA patients at the University Teaching Hospitals, Lusaka, Zambia.

## 2. Materials and Methods

### 2.1. Study Design

The study was a cross-sectional study conducted at the University Teaching Hospital (UTH) in Lusaka, Zambia.

### 2.2. Study Population

The study included both male and female participants of all age groups with sickle cell anaemia who visited the UTH sickle cell clinic between April and July 2019. All patients with a history suggestive of recurrent UTI were excluded from the study.

### 2.3. Sample Collection

A qualified or trained clinical personnel collected blood samples via a vein puncture from each patient for blood cell counts and kidney function tests. Each patient was asked to submit midstream urine in sterile containers for urine culture after being given instructions on how to collect the sample by the attending health personnel. The urine samples were cultured within 2 hours upon receipt. Otherwise, they would be stored at 4°C in a refrigerator for up to 24 hours maximum before culture.

### 2.4. Diagnosis of Sickle Cell Anaemia

Sickle cell anaemia was diagnosed by first running the haemoglobin solubility test. Specimens that showed precipitation in high-molarity-buffered phosphate solution in this test were then confirmed for sickle cell anaemia using gel haemoglobin electrophoresis on the Helena SAS-1 plus and Helena SAS-2 Auto-stainer (Helena Bioscience, Europe).

### 2.5. Full Blood Count and Kidney Function

The Sysmex XT-4000i (Sysmex, Japan) haematology analyser was used to carry out haematological analysis while kidney function was assessed by determining the serum creatinine levels using the Pentra C200 by Horiba (Horiba, Japan). We used creatinine to assess kidney function because studies have shown that serum creatinine above specific cutoff points reliably identifies patients with acute kidney injury or chronic kidney disease [15–17]. All quality control procedures were followed before running the specimens.

### 2.6. Urine Culture

The urine samples were inoculated on blood and Cysteine Lactose Electrolyte Deficient (CLED) agars. Briefly, a well-calibrated loop of 1 *μ*L was dipped in a vertical position in the urine sample, and the loop was used to inoculate the plates using the streak plate method. The urine sample was streaked onto the agar plates by holding the loop at 45° angle to the agar plate. The agar plates were then incubated at 37°C for 24 hrs. The plates of blood agar were incubated in 5–10% CO_2_ atmosphere. After 48 hrs of incubation, the urine cultures were classified as negative (if there was no bacterial growth), positive (if there was a pure bacterial growth of one colony type), and contaminated (if there was a mixed growth of more than three types of bacterial colonies). The samples were classified as contaminated when polymorphic bacterial growth was observed and as insignificant when bacterial growth was lower than 10^3^ colony-forming unit (CFU)/mL and significant when monomorphic bacterial growth was higher than 10^5^ CFU/mL [18]. Biochemical identification of isolates was made on culture-positive samples. These tests were performed based on the morphology of the isolated bacteria and the results of the microscopic examination of the Gram-stained smear. The *Enterobacteriaceae* were differentiated using triple sugar iron, Simmon's citrate, urease, lysine iron , motility, and indole tests. Pure colonies were used for the inoculation of test media and were incubated for 18 to 24 hours at 37°C. For Gram-positive isolates, the catalase test was used to distinguish *Staphylococcus* species from *Streptococcus* species. Furthermore, the coagulase test (Biomérieux, Slider Staph plus) was used to differentiate *Staphylococcus aureus* from the other *Staphylococci*.

### 2.7. Data Analysis

Data were entered in Excel and analysed using Python 3.7 for Mac. Age characteristics, anaemia prevalence and severity, WBC count categories, and UTI prevalence were analysed using descriptive statistics and presented as means, frequencies, percentages, and graphs. Data were checked for normality using the Shapiro–Wilk test and log-transformed to a nearly normal distribution. A one-way analysis of variance (ANOVA) was used to compare the mean difference of haematological parameters across our defined age group categories. Logistic regression analysis was used to examine the relationship between bacteria isolation and age groups, sex, anaemia severity, and white blood cell categories, and a *p* value of <0.05 was used to indicate statistical significance.

### 2.8. Ethical Consideration

Ethical approval for the study was obtained from the University of Zambia Health Sciences Research Ethics Committee (UNZAHSREC) (Protocol ID: 20190217067). Informed consent was obtained from the adults while assent for the children to take part in the study was obtained from their guardians.

## 3. Results

### 3.1. Demographic Characteristics

**Figure 1:**
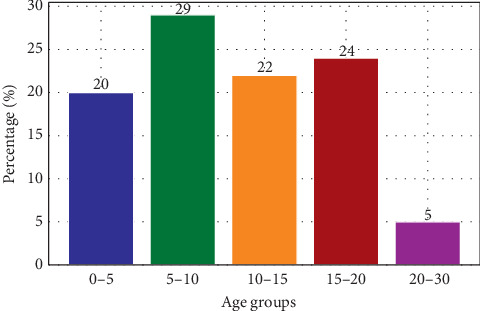
Age distribution of participants.

The study consisted of 78 participants out of which 45 (58%) were females and 33 (42%) were males. The minimum age was 2 years old and the maximum age was 28 years old, with a mean age of 11.4 ± 6.1 years. Participants were divided into age group categories, as shown in Figure 1 below. Sixty-six (86%) were on folic acid and deltaprim, while 11 (14%) were on folic acid only treatment.

### 3.2. Prevalence of Kidney Disease in SCA Patients

Minimum creatinine concentration of the participants was 19 *μ*mol/l and maximum 75 *μ*mol/l with the mean of 38.23 ± 12.3 *μ*mol/l. All participants had creatinine levels below 110 *μ*mol/l.

### 3.3. Haematological Parameters in SCA Patients of Different Age Groups

All patients had Hb levels below the WHO-recommended reference range (11 g/dl for females and 13 g/dl for males) [19] with a minimum of 5 g/dl, maximum of 10.5 g/dl, and a mean of 8 ± 1 g/dl. We found that 50% of the participants had moderate anaemia, whereas the other 50% had severe anaemia with none having mild anaemia. We classified the severity of anaemia based on the World Health Organisation guidelines [19].

Using ANOVA, we showed that there was no significant difference in mean Hb concentration across age groups, *F* (4.67) = 1.316, *p* = 0.273. The minimum WBC count of the participants was 0.02 × 10^9^/L with a maximum of 23.36 × 10^9^/L and a mean of 13.48 ± 3.87 × 10^9^/L. Ninety percent (90%) had high WBC count (10 × 10^9^/L), 7% normal count (between 4 × 10^9^/L and 10 × 10^9^/L), and 3% had low WBC count (below 4 × 10^9^/L Table 1). There was no significant difference in mean WBC counts across age groups using ANOVA, *F* (4.67) = 0.19, *p*=0.466.

### 3.4. Asymptomatic UTIs in Different Age Groups of SCA Patients

**Figure 2:**
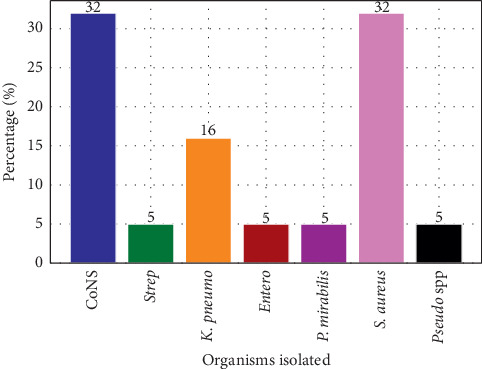
Organisms isolated from urine cultures. CoNS: coagulase-negative *Staphylococci*, *Strep*: *Streptococcus*, *K pneumo*: *Klebsiella pneumoniae*, *Entero*: *Enterococcus*, *P mirabilis*: *Proteus mirabilis*, *S aureus: Staphylococcus aureus*, *Pseudo* spp: *Pseudomonas* species.

Out of 72 urine specimens cultured, we found growth of bacteria in 18 (25%) specimens, whereas the other 54 (75%) specimens had no growth (Figure 2). The most common bacterial isolates were *Staphylococcus aureus* (32%) and coagulase-negative *Staphylococci* (32%). *Klebsiella pneumoniae* was 16%, *Enterococcus* 5%, *Streptococcus* 5%, *Pseudomonas* species 5%, and *Proteus mirabilis* were 5%.

We performed logistic regression analysis to examine the relationship between bacterial isolation and white blood cell count categories using age groups, sex, and anaemia severity as possible confounders. Here, our results revealed a nonstatistically significant (*p*=0.41) relationship between bacterial isolation and all the variables.

## 4. Discussion

We found that all participants had normal kidney function. The low and normal serum creatinine levels that we found might have been due to hyperfiltration of creatinine that is reported in most young SCA patients [11]. In patients with SCA, the glomerular filtration rate (GFR) begins to decline in the second decade of life [20]. This may, in part, explain why we found normal serum creatinine levels as most of our study participants were in their first decade of life. Others have reported kidney disease in SCA, albeit at very low prevalence of 6% [21]. The difference can be attributed to the sample population that was used in the study in Jamaica which only included participants who were in their fourth decade of life. However, the normal creatinine levels found in our study were similar to the creatinine levels that were found in a study that was done in Saudi Arabia which concluded that serum creatinine might remain low or within the low-normal range in SCA patients despite the reduced creatinine clearance [22]. The serum creatinine can remain within the normal range because it only begins to decline after the GFR has reduced to about 50 ml/min, and many years are taken to reach this GFR.

All the participants had haemoglobin levels below normal concentration. The low haemoglobin levels might have been due to haemolysis of red cells consequent to the damaged red cell membrane. In SCA, the haemolysis of red cells is intravascular or extravascular. Intravascular haemolysis results from the lysis of complement sensitive red cells and haemoglobin lost during sickling-induced membrane damage [23]. Extravascular haemolysis occurs by phagocytosis of red cells that have undergone sickling and physical entrapment of compromised red cells. The low haemoglobin levels in SCA patients that we found in this study was similar to the findings in a study that was done in Nigeria in which the mean haemoglobin level of 103 SCA patient participants was 7.93 ± 1.47 g/dl [24]. Here also, the shortened red cell survival and lowered erythropoietin response that is associated with SCA have a bearing towards the development of anaemia in SCA [10].

We found a high WBC count in the majority of the participants similar to other reports [24, 25]. Recurrent infection is a known predisposing factor to sickle cell disease crises and is associated with leukocytosis [26]. High WBC count has also been associated with severe anaemia [24]. Therefore, the high WBC count reported here might in part explain the presence of moderate to severe anaemia in all of the participants in the study. The findings corroborate with the findings of a study that was done in Saudi Arabia in which SCD participants who had high WBC count were also diagnosed with severe anaemia [25]. The high WBC count is a serious concern for sickle cell patients as it is associated with shortness of breath, tiredness, swelling in hands/feet, and back pain [25]. High WBC counts have also been found to be a risk factor for early SCD-related death [5], clinically overt stroke [27, 28], silent cerebral infarction [29], and acute chest syndrome [30].

The proportion of SCA patients with asymptomatic UTIs (25%) that we found was higher than those reported in Nigeria (6%); [31] and in Jamaica (5.3%) [32]. Since the prevalence of asymptomatic UTIs increases with age, these results are expected as the participants in our study were older than those of the Jamaican and Nigerian studies whose participants were between the ages of 2–12 years.

We found that the aetiologic pathogens associated with UTIs in our study included *Klebsiella* spp, *Staphylococcus aureus*, *Streptococci*, *Enterococcus* spp, *Pseudomonas*, *Proteus mirabilis*, and *E*. *coli* which are the major causes of UTIs worldwide. Additionally, organisms such as *Staphylococcus aureus*, *Streptococcus*, and *Proteus* have a broad representation as causative agents of UTIs in developing countries due to poor environment and personal hygiene [31]. Therefore, environmental factors and poor hygiene cannot be ruled out as contributing factors towards our report of *Staphylococcus aureus* (24%), *Streptococcus* (4%), and *Proteus* (4%) in the urine of SCA patients. Furthermore, impairment of the immune system in SCA also contributes towards the presence of asymptomatic bacteriuria, including infections with *Proteus* and *Staphylococcus species* [31]. With all these findings of our study, we recommend that the Zambia ministry of health introduces a newborn screening for sickle cell program using techniques such as dry blood spots [33]. The introduction of such a program may help improve the quality of life of sickle cell patients as they will be able to receive proper health care from childhood.

### 4.1. Limitations

There is a possibility that some participants have early kidney malfunction, which may not be detected by the use of serum creatinine [34]. Serum creatinine is considered a late marker of kidney injury since a rise in creatinine can only occur after about 50% of kidney function is lost [35].

## 5. Conclusion

There is a need for close monitoring of sickle cell patients in Zambia as there is a high prevalence (25%) of asymptomatic UTIs, and the majority of them (90%) has a high WBC count which impacts negatively on the clinical outcome of the patients. Also, we recommend the introduction of newborn screening for SCA so that the patients can receive the right treatment from the time they are still young.

## Figures and Tables

**Table 1:** Percentage of white blood cell categories.

WBC count	Percentage (%) of participants
Low	3
Normal	7
High	90

## Data Availability

The data used to support the findings of this study are available from the corresponding author upon request.
